# Perforated jejunal GIST after blunt abdominal trauma: a case report

**DOI:** 10.1093/jscr/rjag739

**Published:** 2026-08-24

**Authors:** Bianca Marquez, Akram Alashari

**Affiliations:** Department of Surgery, Covenant Healthcare Central Michigan University College of Medicine, Saginaw, MI 48601, United States; Department of Surgery, Covenant Healthcare Central Michigan University College of Medicine, Saginaw, MI 48601, United States

**Keywords:** GIST, small bowel, blunt trauma, perforation

## Abstract

We present a rare case of perforated jejunal gastrointestinal stromal tumour (GIST) following minor blunt abdominal trauma. Small bowel GISTs are uncommon entities, and presentation with acute perforation and generalized peritonitis is particularly rare, especially in the absence of prior diagnosis or significant inciting trauma. A 40-year-old male presented with an acute abdomen and clinical peritonitis consistent with a perforated viscus. Laboratory evaluation revealed significant leukocytosis, and cross-sectional imaging demonstrated pneumoperitoneum, prompting urgent surgical intervention. Given the combined clinical, radiologic, and laboratory findings, the patient underwent emergent exploratory laparotomy with segmental small bowel resection. This case highlights an uncommon presentation of jejunal GIST and underscores the importance of prompt recognition of peritonitis, timely operative management, and early multidisciplinary oncologic evaluation following definitive surgical treatment.

## Introduction

Gastrointestinal tumours (GISTs) are one of the most common mesenchymal tumors originating from the interstitial cells of Cajal of the GI tract. Clinical presentation is known to be variable but may include abdominal pain, GI bleeding, obstruction, and/or even rarely perforation. The most common locations include the stomach (60%–70%) and second most common location is the small bowel (25%–30%) [1]. Definitive diagnosis is established through histopathological examination. GISTs typically demonstrate spindle cell, epithelioid, or pleomorphic morphology, with small bowel lesions most commonly exhibiting spindle cell features. Immunohistochemical staining is essential for confirmation, with most tumours expressing KIT (CD117) or PDGFRA mutations in CD117-negative cases [2, 3]. This case report presents the acute surgical management in a case of a perforated jejunal GIST after mild blunt abdominal trauma.

## Case presentation

**Figure 1:**
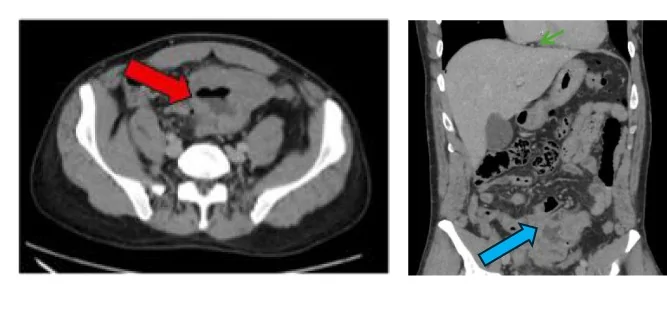
Axial (right) CT of the abdomen and pelvis showing the small bowel with associated abscess 4.5 × 3.3 cm (red arrow). Coronal (left) CT of abdomen and pelvis showing pneumoperitoneum (green arrow) and thickened/distended loops of small bowel with indeterminate mass in the mid abdomen (blue arrow).

A 40-year-old male with no significant past medical history presented with acute onset severe abdominal pain following mild blunt abdominal trauma sustained 1 day prior to admission. The injury occurred when another individual landed directly on his abdomen with significant force. On initial evaluation, the patient demonstrated abdominal distention with peritonitis on examination. Patient remained afebrile and hemodynamically stable. Laboratory studies were notable for marked leukocytosis with a white blood cell count of 23.7 × 10^9^/L. Computed tomography (CT) of the abdomen and pelvis with intravenous contrast demonstrated pneumoperitoneum with an associated 4.5 × 3.3 cm interloop abscess involving the mid jejunum, concerning for hollow viscus perforation (Fig. 1).

**Figure 2:**
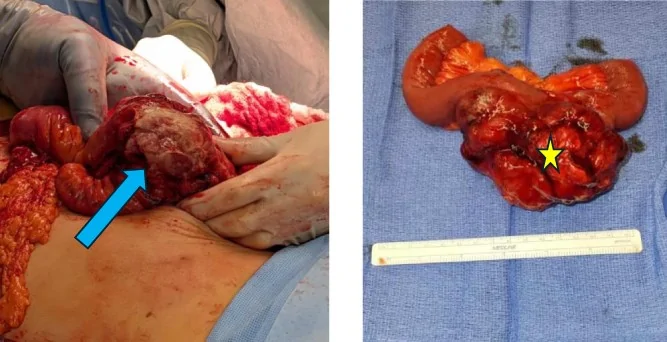
Intraoperative images showing a jejunal mass with small perforation (blue arrow), and resected jejunum with perforated mass along the antimesenteric border (yellow star).

Given the clinical and radiographic findings, the patient was taken emergently to the operating room for exploratory laparotomy. Upon entering the peritoneal cavity, inflamed small bowel with associated desmoplastic reaction was immediately encountered. Further exploration identified a large exophytic jejunal mass measuring ~9 cm arising from the antimesenteric border of the mid jejunum. The lesion was grey–tan, firm, and locally inflammatory in appearance (Fig. 2). Inspection revealed focal perforation of the tumour capsule with associated purulent contamination, confirming the etiology of the pneumoperitoneum.

A segmental small bowel resection encompassing the involved jejunal segment was performed with adequate gross margins, followed by primary stapled side-to-side functional end-to-end anastomosis in standard fashion. The remainder of the small bowel was systematically examined from the ligament of Treitz to the terminal ileum without evidence of additional pathology. Incidentally, the appendix was noted to be dilated and inflamed without evidence of perforation; therefore, appendectomy was performed. All specimens were submitted for permanent histopathologic evaluation. The patient tolerated the procedure without intraoperative complication.

**Figure 3:**
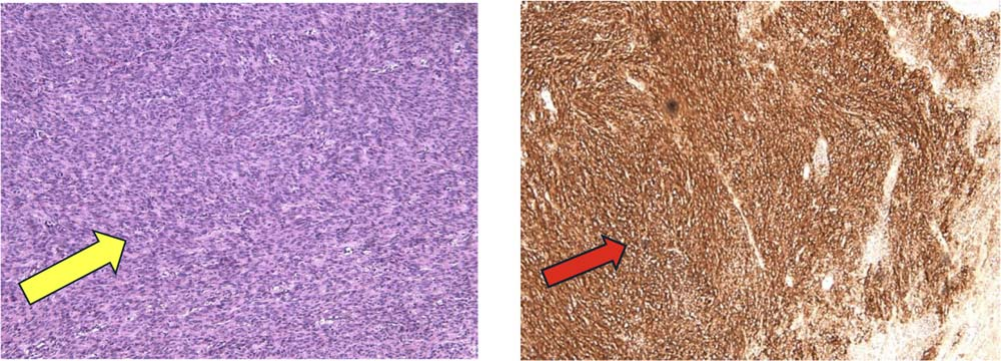
Haematoxylin and eosin stain (right) pathologic slide of dense proliferation of fascicles of spindle cells (yellow arrow). CD117 stain (left) pathologic slide pathologic slide showing diffuse CD117 positivity. Red arrow showing the uptake within the cytoplasm of the spindle cells.

Postoperatively, the patient recovered appropriately with an uncomplicated hospital course and was discharged on postoperative Day 5. Final pathology demonstrated a high-grade jejunal GIST, Grade 2, with a mitotic rate of 15 mitoses per 5 mm^2^, staged pT3Nx (Fig. 3). At postoperative follow-up, the patient was recovering well and had initiated adjuvant therapy with Imatinib.

## Clinical discussion

GISTs are the most common mesenchymal neoplasms of the gastrointestinal tract and are characterized by activating mutations in KIT or PDGFRA. The stomach is the most frequent site of origin (~70%), while the jejunum accounts for roughly 10% of cases. Clinical presentation is variable and may include vague abdominal pain, gastrointestinal bleeding, obstipation, or, rarely, peritonitis secondary to tumour perforation [4]. Jejunal GISTs may additionally present with nonspecific symptoms such as abdominal pain, early satiety, a palpable mass, and abdominal fullness [1]. Tumour perforation following blunt abdominal trauma is uncommon but clinically significant due to its association with increased morbidity and mortality [5, 6].

Preoperative diagnosis of perforated GIST remains challenging. In the present case, CT demonstrated pneumoperitoneum with a large associated intra-abdominal abscess. In the context of the patient’s clinical presentation, these findings prompted urgent operative exploration. Although CT is the most widely available imaging modality in the acute setting, magnetic resonance imaging has been shown to provide superior soft-tissue characterization of GISTs [7]. Definitive diagnosis, however, relies on histopathological examination following surgical resection. Previous reports have described jejunal GISTs presenting with interloop or intramural abscess formation in the setting of perforation, requiring emergent surgical management [8, 9]. Mohamed et al. reported a rare case of gastric GIST rupture resulting in haemorrhagic shock following blunt abdominal trauma [10]. These cases highlight the rarity and diagnostic difficulty of perforated small bowel GISTs and reinforce the importance of prompt surgical intervention.

The mainstay of treatment for localized GIST is complete surgical resection with negative margins. In this case, given the presence of a large abscess cavity and jejunal perforation, an en bloc small bowel resection was performed [3, 8, 11]. Adjuvant therapy with the tyrosine kinase inhibitor imatinib is indicated in high-risk disease and has been shown to improve recurrence-free and overall survival, particularly in cases with tumor perforation, high-grade pathology, or metastatic potential. Accordingly, the patient was commenced on adjuvant imatinib given the high-risk features and histopathological evidence of perforation.

## Conclusion

Jejunal GISTs are rare and typically present with non-specific symptoms, which can lead to a broad differential diagnosis. However, perforated jejunal GISTs complicated by abscess formation following blunt trauma are associated with significantly increased morbidity and mortality. This case highlights the importance of maintaining a high index of suspicion and emphasizes the need for early diagnosis and urgent surgical intervention in patients presenting after even minor trauma with imaging findings suggestive of small bowel abscess and pneumoperitoneum.
